# Continue or not to continue? Attitudes towards deprescribing among community-dwelling older adults in China

**DOI:** 10.1186/s12877-022-03184-3

**Published:** 2022-06-08

**Authors:** Jie Tan, MinHong Wang, XiaoRui Pei, Quan Sun, ChongJun Lu, Ying Wang, Li Zhang, Chenkai Wu

**Affiliations:** 1 Global Health Research Center, Duke Kunshan University, Academic Building 3038 No. 8 Duke Avenue, 215316 Kunshan, Jiangsu China; 2 Department of Geriatric Medicine, Suzhou Municipal Hospital, Gusu School, The Affiliated Suzhou Hospital of Nanjing Medical University, Nanjing Medical University, 215002 Suzhou, Jiangsu Province China; 3 Gusu District Wumenqiao street Nanhuan community Health Service Center, 215008 Suzhou, Jiangsu Province China; 4 Gusu District Pingjiang street Loujiang community Health Service Center, 215008 Suzhou, Jiangsu Province China

**Keywords:** Deprescribing, Polypharmacy, Older adults, China, Patient attitudes, Potentially inappropriate medications (PIMs)

## Abstract

**Background:**

Inappropriate prescribing of medications and polypharmacy among older adults are associated with a wide range of adverse outcomes. It is critical to understand the attitudes towards deprescribing—reducing the use of potentially inappropriate medications (PIMs)—among this vulnerable group. Such information is particularly lacking in low - and middle-income countries.

**Methods:**

In this study, we examined Chinese community-dwelling older adults’ attitudes to deprescribing as well as individual-level correlates. Through the community-based health examination platform, we performed a cross-sectional study by personally interviews using the revised Patients’ Attitudes Towards Deprescribing (rPATD) questionnaire (version for older adults) in two communities located in Suzhou, China. We recruited participants who were at least 65 years and had at least one chronic condition and one prescribed medication.

**Results:**

We included 1,897 participants in the present study; the mean age was 73.8 years (SD = 6.2 years) and 1,023 (53.9%) were women. Most of older adults had one chronic disease (*n* = 1,364 [71.9%]) and took 1–2 regular drugs (*n* = 1,483 [78.2%]). Half of the participants (*n* = 947, 50%) indicated that they would be willing to stop taking one or more of their medicines if their doctor said it was possible, and 924 (48.7%) older adults wanted to cut down on the number of medications they were taking. We did not find individual level characteristics to be correlated to attitudes to deprescribing.

**Conclusions:**

The proportions of participants’ willingness to deprescribing were much lower than what prior investigations among western populations reported. It is important to identify the factors that influence deprescribing and develop a patient-centered and practical deprescribing guideline that is suitable for Chinese older adults.

**Supplementary Information:**

The online version contains supplementary material available at 10.1186/s12877-022-03184-3.

## Background

Potentially inappropriate medications (PIMs) can lead to adverse drug events, drug interactions, falls, fractures, functional or cognitive impairment, poor adherence, hospitalization, and excessive medical costs, especially in older patients with comorbidities [1–5]. To limit such risks, deprescribing, which is defined as a patient-centered strategy of drug withdrawal aimed at improving health outcomes by discontinuing prescriptions that are either hazardous or no longer required [6], has been proposed as a method of addressing concurrent use of multiple medications [7]. It is a consensus that patients should be engaged in the prescribing process since patient-centered treatment could optimize health outcomes [8, 9]. Therefore, having a better understanding of the attitudes towards medication use and willingness to deprescribe among older adults would be one of the necessary steps to implement deprescribing in geriatric practice.

The revised Patients’ Attitudes Towards Deprescribing (rPATD) questionnaire was recently developed to assess patients’ perceptions about medication use and deprescribing [10]. To date, rPATD studies have been conducted across a number of countries including Australia [11], Singapore [12], Ethiopia [13], Malaysia [14], Netherlands [15], United States [16] and United Kingdom [17]. Studies conducted in different countries have shown that the acceptance rates of deprescribing range from 29% [17] to 93% [18].

Though many western countries have adopted the deprescribing approach, evidence on deprescribing is lacking from developing countries, suggesting the concept is yet novel [19, 20]. Moreover, no previous study has explored the older patients’ attitudes toward deprescribing in China, which has the largest older population in the world and is expected to experience the fastest growth of population aging in the next 20 years [21]. This study aimed to identify Chinses community-dwelling older adults’ attitudes and beliefs towards deprescribing by rPATD questionnaire, and to explore socio-demographic and lifestyle factors for attitudes towards deprescribing.

## Methods

### Study design, setting and participants

This cross-sectional study was conducted in two community health centers in Suzhou, China from October 2020 to April 2021 (Wumen Bridge South Ring Community and Loujiang Community). All older residents (at least 65 years) living in the above-mentioned two communities were invited to participate in regular physical examinations, during which blood and urine samples and self-administered health questionnaires were collected. All these individuals were screened by trained nurses for eligibility to participate in our study. Eligibility criteria include: (1) no significant cognitive impairment, (2) no terminal illness with a life expectancy less than 12 months, and (3) willing to participate. A face-to-face interview was conducted by at the physical examination site for each participant to collect survey data. Four nurses from Suzhou Municipal Hospital conducted all interviews. All participants were given informed consent prior to being enrolled in the study.

A total of 2778 older individuals participated in the questionnaire survey. Of these participants, we included 2733 participants who were 65 years and over, had one or more chronic conditions and took one or more prescription medications.

### Deprescribing attitudes

Older adults’ attitudes towards deprescribing were assessed by eight questions from the validated rPATD questionnaire (version for older adults) [10, 16]. The rPATD questionnaire was developed to measure individuals’ attitudes, beliefs, and experiences with their medications and willingness to medication discontinuation [10]. The eight questions are as follow: (1) If my doctor said it was possible, I would be willing to stop one or more of my regular medicines; (2) I would like to reduce the number of medicines I am taking; (3) I have a good understanding of the reasons I am taking each of my medicines; (4) I believe that all of my medicines are necessary; (5) I would be willing to stop a medicine that I have been taking for a long time; (6) I feel that I am taking a large number of medicines; (7) I get stressed whenever changes are made to my medicines; (8) I feel that I may be taking one or more medicines that I no longer need. The response options were modified from the rPATD to a 4-point Likert scale, and four response options were available for each of these eight statements: “highly agree,“ “agree,“ “disagree,“ and “highly disagree.“ The results are self-reported by the participants. In this study, the rPATD questionnaire was first translated into Chinese by two independent bilingual translators and back to English to ensure that the translated version gave the proper meaning.

### Other variables

During the interview, in addition to participants’ attitudes towards deprescribing, data on sociodemographic characteristics such as participants’ age, gender, the highest level of education attained, marital status, living arrangements, financial assistance, and clinical characteristics such as height, weight, and blood pressure were also captured.

### Statistical analysis

Data were entered into Epidata 4.6.0.6 and analyzed in Stata 16.0. Participants’ characteristics and rPATD responses were reported using descriptive statistics. Categorical variables were reported as frequency and percentage, and continuous variables were reported as mean and standard deviation. Participants’ responses to the rPATD questions were dichotomized to agree (highly agree or agree) or disagree (disagree or highly disagree). Similar to a previous study by Reeve et al. [16], two of the scale items were chosen as the major outcomes of interest because they gave an overview of the individual’s attitudes to deprescribing. “If my doctor said it was possible, I would be willing to stop one or more of my regular medicines” and “I would like to reduce the number of medicines I am taking,“ are referred to as “willingness to discontinue” and “wanting to reduce,“ respectively [16]. We excluded participants with any missing item of the rPATD questionnaire. To assess the association between those who completely answered the questionnaire and who didn’t, the bivariate analyses were conducted. We used logistic regression to analyze unadjusted correlations between the two primary statements and respondents’ demographic and clinical characteristics. After adjusting for demographic and clinical factors, multivariable regression models were used to investigate the likelihood of willingness to discontinue and wanting to reduce. *P *⩽0.05 was considered statistically significant.

## Results

### Sample characteristics

Among the recruited 2733 participants, we excluded 836 participants who didn’t completely answer the rPATD questionnaire. Table S1 showed that there were not huge differences between the included and excluded participants. Of the 1,897 eligible participants, the median age was 73.8 years; 29.6% (*n* = 561) were 65–69 years, 30.3% (*n* = 575) were 70–74 years, 20.6% (*n* = 392) were 75–79 years, and 19.5% (*n* = 369) were at least 80 years (Table 1). There were 1,023 females (53.9%). Nearly 80% were married and living with a spouse and slightly over 85% lived with family members; only 27.0% (*n* = 509) reported having attended high school education and above. The three most common chronic conditions were hypertension (90.1%), diabetes mellitus (24.7%) and hyperlipidemia (5.8%). Approximately 6% had three or more chronic medical conditions, and 21.8% took three or more regular medications. Moreover, 6.5% of the participants reported fair or poor self-rated health.

**Table 1:** Demographic and clinical characteristics of the study population

Demographic characteristics	Total *N* = 1897 (%)
**Age, mean (SD)**	73.8 (6.2)
**Age group**	
65–69	561 (29.6)
70–74	575 (30.3)
75–79	392 (20.6)
≥ 80	369 (19.5)
**Sex**	
Men	874 (46.1)
Women	1023 (53.9)
**Married/living with spouse vs. others**	1457 (77.0)
**Highest educational level**	
Primary school and below	571 (30.4)
Middle school	801 (42.6)
High school and above	509 (27.0)
**Living with family vs. along**	1641 (86.6)
**Medical conditions**	
Hypertension	1709 (90.1)
Diabetes	469 (24.7)
Hyperlipidemia	109 (5.8)
Coronary heart disease	95 (5.0)
Gout	13 (0.7)
Chronic obstructive pulmonary disease	9 (0.5)
Stroke	53 (2.8)
Rheumatism	7 (0.4)
Arthritis	7 (0.4)
Cancer	7 (0.4)
Others	81 (4.3)
**Number of chronic conditions**	
1	1364 (71.9)
2	421 (22.2)
≥ 3	112 (5.9)
**Number of medications**	
1–2	1483 (78.2)
≥ 3	413 (21.8)
**Self-rated health**	
Excellent/good	1774 (93.5)
Fair/poor	123 (6.5)

### Older adults’ beliefs and attitudes toward deprescribing

**Fig. 1:**
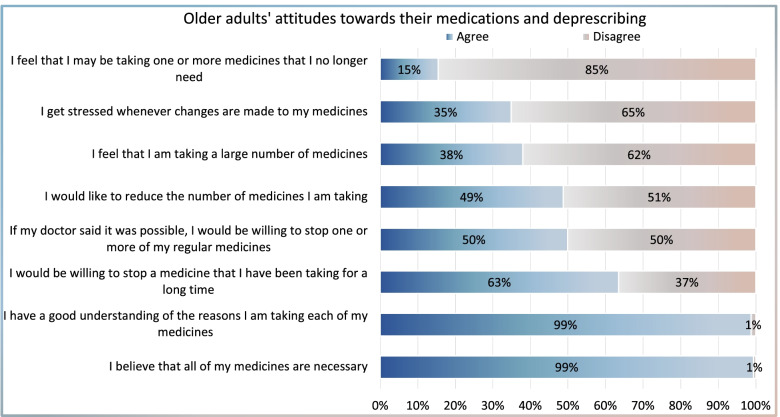
Participants responses to the revised Patients’ Attitudes Towards Deprescribing (rPATD) questionnaire

Participants’ responses to the rPATD questionnaire were presented in Fig. 1. Nearly all participants thought they had a good understanding of the reasons why they were taking these medicines (98.8%) and believed that all their medications were necessary (99.4%). There were 48.7% of the participants had the desire to reduce the number of medications they were taking, and half of the participants reported that they would be willing to stop one or more of their medicines if their physician said it was possible. In addition, 63.5% of the participants would be willing to stop a medicine they have been taking for a long time. Moreover, 38.0% considered that they were taking many medications, 15.5% felt that they were taking medications that they no longer needed, and 34.8% of the respondents reported that they got stressed whenever changes were made to their medicines.

As shown in Table 2, there were no demographic and clinical characteristics associated with willingness to stop and wanting to reduce. An adjusted multivariate analysis for all the eight questions was shown in the supplementary Table 2.

**Table 2:** Unadjusted and adjusted associations between older adults’ demographic and clinical characteristics and their attitudes toward deprescribing

	If my doctor said it was possible, I would be willing to stop one or more of my regular medicines, OR (95% CI)		I would like to reduce the number of medicines I am taking, OR (95% CI)	
**Characteristics**	**Unadjusted**	**Adjusted**	**Unadjusted**	**Adjusted**
**Age**				
65–69	ref.	ref.	ref.	ref.
70–74	1.03 (0.82–1.30)	1.01 (0.80–1.28)	0.95 (0.76–1.20)	0.93 (0.74–1.18)
75–79	1.01 (0.78–1.30)	0.99 (0.75–1.29)	0.91 (0.70–1.18)	0.88 (0.67–1.15)
≥ 80	1.17 (0.90–1.52)	1.15 (0.87–1.53)	1.00 (0.77–1.30)	0.97 (0.73–1.28)
**Sex**				
Male	ref.	ref.	ref.	ref.
Female	1.02 (0.85–1.23)	1.03 (0.85–1.25)	1.04 (0.87–1.25)	1.02 (0.84–1.23)
**Marital status**				
Married/living with spouse	ref.	ref.	ref.	ref.
others	1.02 (0.82–1.26)	1.00 (0.72–1.38)	1.08 (0.87–1.34)	1.08 (0.78–1.50)
**Education**				
Primary school and below	ref.	ref.	ref.	ref.
Middle school	0.88 (0.71–1.09)	0.90 (0.72–1.13)	0.93 (0.75–1.15)	0.95 (0.76–1.19)
High school and above	0.94 (0.74–1.19)	0.98 (0.76–1.26)	0.91 (0.72–1.16)	0.95 (0.74–1.23)
**Living arrangement**				
Living with family	ref.	ref.	ref.	ref.
alone	0.99 (0.76–1.29)	0.97 (0.66–1.42)	1.05 (0.81–1.37)	0.98 (0.67–1.44)
**Number of chronic conditions**				
1	ref.	ref.	ref.	ref.
2	1.05 (0.85–1.31)	0.99 (0.72–1.37)	1.12 (0.90–1.40)	1.09 (0.79–1.51)
≥ 3	1.01 (0.69–1.49)	0.98 (0.60–1.61)	1.26 (0.86–1.86)	1.29 (0.79–2.11)
**Number of medications**				
1–2	ref.	ref.	ref.	ref.
≥ 3	1.06 (0.86–1.32)	1.07 (0.75–1.51)	1.14 (0.92–1.42)	1.03 (0.72–1.46)
**Self-rated health**				
Excellent/good	ref.	ref.	ref.	ref.
Fair/poor	0.80 (0.55–1.15)	0.77 (0.53–1.13)	0.71 (0.49–1.02)	0.69 (0.47–1.01)

## Discussion

### Summary and comparison with existing literature

Overall, we found that although almost all participants had a firm belief in the necessity of their medications and thought they had a good understanding of the medicines they were taking, still half of them were willing to stop one or more of their medications if their doctor said it was possible, and almost half of them expressed willingness of reducing the number of medicines they were taking. These results concur with previous rPATD studies which demonstrated that the majority of participants agreed with deprescribing proposed by a doctor whilst also be satisfied with current medicines [22–25].

We found that 50% of community-dwelling older adults in China were willing to deprescribing, which was lower than that in other Asian and western countries. In Asian countries, Kua KP et al. [14] found that 67.7% of older adults in Malaysia and Kua CH et al. [12] found that 83.0% of older adults in Singapore were willing to stop one or more of their medications if their doctor said it was possible. The level of older adults’ willingness to deprescribing was generally higher in western countries such as 92% in the United States [16], 88% in Australia [26], and 87% in Denmark [27]. Also, a recent systematic review suggested that 84% of older adults were willing to deprescribe [28]. These differences may be explained by the fact that participants in our study were in better health. Indeed, nearly 80% of patients in the current study took fewer than three medications, and older adults in Kua KP et al. study were taking a median of three medications, while the median number of patients’ daily medications was 11, 8, and 6 in Reeve et al. [18], Carina et al. [27] and Sirois et al. studies [29] respectively.

One plausible explanation for the relatively low willingness to deprescribing in the present study is that physicians in China may have a strong influence on older adults’ attitudes towards medication use and deprescribing [30]. Patients in China are more likely to conform to physicians’ recommendations despite concerns about their medications [31, 32]. Meanwhile, Chinese physicians still tended to use complicated and long-term medication regimens in treatment [32]. Moreover, the proportion of older adults taking traditional Chinese medicine (TCM) was high in China and older adults have a high adherence to TCM [31, 33]. This might help explain why Chinese older adults were less willing to reduce medication use than their Western counterparts. Besides, fear, low health literacy, time limitations, professional hindrances, and belief that their medicines are appropriate are also barriers to deprescribing in practice [26, 34–36].

We did not find age, the number of medications uses, or the number of medical conditions to be associated with older adults’ willingness to stop a medication. This is consistent with Oktora et al. systematic review [20] which declared that the patients’ sex or education were not associated with their attitudes toward deprescribing generally and age was not associated with their attitudes at individual level. The current investigation could not identify characteristics that could select individuals who are more favorable toward deprescribing. This implies that to provide patient-centered care, all residents should be evaluated individually for deprescribing, regardless of how many medications they are taking or how old they are.

### Strengths and limitations

This study is among the first to use a validated multidimensional questionnaire to measure older individuals’ willingness to deprescribing in China. We acknowledged several limitations. First, as only a portion of the rPATD questionnaire was used, factor scores could not be calculated. In addition, the rPATD questionnaire was originally designed in English and had not been validated in China previously. Moreover, our findings need to be interpreted cautiously because our study was conducted in one study site, which may not be representative of the entire Chinese older population. Lastly, as the participation was based on volunteers, acceptance rates in studies may be biased by individuals who are willing to consent to study participation.

### Implications for clinical practice and future research

Although the willingness of deprescribing among community-dwelling older adults is relatively low in this study, practitioners should not dismiss deprescribing opportunities due to the benefits of deprescribing such as increasing the patient’s engagement in medication therapy management and improving adherence possibly through reducing polypharmacy [13, 37]. Currently, the guideline for deprescribing have been developed in Australia [4, 38, 39]. We should adapt this guideline to the current situation in China and draw up a patient-centered and practical deprescribing guideline that is suitable for Chinese older adults.

Further research uses complete rPATD questionnaire and conducted with representative sample is needed. Studies that explore the predictors of older adults’ attitudes towards deprescribing is also required. Furthermore, to ascertain the safety and efficacy of the deprescribing, feasibility and implementation research will be needed, and long-term outcomes should be determined while taking economic factors into account.

## Conclusions

This study suggests that half of the community-dwelling older adults were willing to cease a medication if their physician thought possible, and almost half of them would like to reduce the number of medicines they were taking. The attitudes toward deprescribing were not associated with sex, age, number of medications, or number of chronic diseases. Future studies with more advanced method and more representative samples were needed. Additionally, we should adapt the guideline developed in Australia to the current situation in China and draw up a patient-centered and practical deprescribing guideline that is suitable for Chinese older adults.

## Supplementary Information

**Additional file 1:** **Supplement Table 1.**Comparison between included and excluded participants. **Supplement Table 2. **Adjusted associationsbetween older adults’ demographic and clinical characteristics and theirattitudes toward deprescribing. **Supplementary Table 3.**STrengthening the Reporting of OBservational studies in Epidemiology (STROBE)checklist for cross-sectional studies.

## Data Availability

The datasets generated and analyzed during the current study are not publicly available as this would be in conflict with the informed consent given by the participants but are available from the corresponding author on reasonable request.

## Abbreviations

PIMs: Potentially Inappropriate Medications
rPATD: revised Patients’ Attitudes Towards Deprescribing
TCM: traditional Chinese medicine
