# Supplementary material for: Continue or not to continue? Attitudes towards deprescribing among community-dwelling older adults in China
Source: BMC Geriatr. 2022 Jun 8;22:492. doi: 10.1186/s12877-022-03184-3 (PMC9175377; doi:10.1186/s12877-022-03184-3)
Supplement: Supplementary file 1 — Additional file 1: Supplement Table 1.Comparison between included and excluded participants. Supplement Table 2. Adjusted associationsbetween older adults’ demographic and clinical characteristics and theirattitudes toward deprescribing. Supplementary Table 3.STrengthening the Reporting of OBservational studies in Epidemiology (STROBE)checklist for cross-sectional studies. [file 12877_2022_3184_MOESM1_ESM.docx]

**Supplement Table 1. Comparison between included and excluded participants.**

| Demographic characteristics | Total  N=2733 (%) | Included participants  N=1897 (%) | Excluded participants  N=836 (%) | P value |
| --- | --- | --- | --- | --- |
| **Age, mean (SD)** | 73.5 (6.2) | 73.8 (6.2) | 72.6 (6.1) | .006 |
| **Female** | 1489 (54.5) | 1023 (53.9) | 466 (55.7) | .380 |
| **Married/living with spouse(others)^a^** | 2106 (77.2) | 1457 (77.0) | 649 (77.7) | .664 |
| **Highest educational level** |  |  |  | .077 |
| Primary school and below | 793 (29.2) | 571 (30.4) | 222 (26.7) |  |
| Middle school | 1157 (42.7) | 801 (42.6) | 356 (42.8) |  |
| High school and above | 763 (28.1) | 509 (27.1) | 254 (30.5) |  |
| **Living with family vs along** | 2373 (86.9) | 1641 (86.5) | 732 (87.7) | .427 |
| **Self-rated health** |  |  |  | .109 |
| Excellent/good | 2569 (94.0) | 1774 (93.5) | 795 (95.1) |  |
| Fair/poor | 164 (6.0) | 123 (6.5) | 41 (4.9) |  |

**^a^:** Other categories include married/ not living with spouse; divorce; widowed; unmarried.

**Supplement Table 2. Adjusted associations between older adults’ demographic and clinical characteristics and their attitudes toward deprescribing**

|  | If my doctor said it was possible, I would be willing to stop one or more of my regular medicines | I would like to reduce the number of medicines I am taking | I have a good understanding of the reasons I am taking each of my medicines | I believe that all of my medicines are necessary | I would be willing to stop a medicine that I have been taking for a long time | I feel that I am taking a large number of medicines | I get stressed whenever changes are made to my medicines | I feel that I may be taking one or more medicines that I no longer need |
| --- | --- | --- | --- | --- | --- | --- | --- | --- |
| **Age** |  |  |  |  |  |  |  |  |
| 65-69 | ref. | ref. | ref. | ref. | ref. | ref. | ref. | ref. |
| 70-74 | 1.01 (0.80-1.28) | 0.93 (0.74-1.18) | 3.00 (0.65-13.77) | 4.06 (0.40-41.04) | 0.90 (0.70-1.16) | 1.07 (0.84-1.37) | 1.09 (0.85-1.39) | 1.02 (0.73-1.41) |
| 75-79 | 0.99 (0.75-1.29) | 0.88 (0.67-1.15) | 0.74 (0.22-2.53) | 0.86 (0.16-4.67) | 0.80 (0.60-1.06) | 1.01 (0.77-1.33) | 1.02 (0.77-1.36) | 0.90 (0.62-1.30) |
| ≥80 | 1.15 (0.87-1.53) | 0.97 (0.73-1.28) | 1.16 (0.31-4.35) | 0.73 (0.13-4.14) | 0.70 (0.52-0.94) | 0.96 (0.72-1.29) | 1.05 (0.78-1.42) | 0.84 (0.56-1.24) |
| **Sex** |  |  |  |  |  |  |  |  |
| Male | ref. | ref. | ref. | ref. | ref. | ref. | ref. | ref. |
| Female | 1.03 (0.85-1.25) | 1.02 (0.84-1.23) | 0.68 (0.24-1.93) | 1.26 (0.31-5.07) | 0.85 (0.69-1.04) | 0.92 (0.75-1.12) | 0.97 (0.79-1.18) | 0.94 (0.72-1.23) |
| **Education** |  |  |  |  |  |  |  |  |
| Primary school and below | ref. | ref. | ref. | ref. | ref. | ref. | ref. | ref. |
| Middle school | 0.90 (0.72-1.13) | 0.95 (0.76-1.19) | 1.11 (0.38-3.24) | 0.87 (0.18-4.27) | 0.74 (0.59-0.95) | 0.91 (0.72-1.16) | 0.94 (0.74-1.19) | 1.00 (0.73-1.38) |
| High school and above | 0.98 (0.76-1.26) | 0.95 (0.74-1.23) | 1.12 (0.33-3.81) | 0.92 (0.16-5.31) | 0.87 (0.67-1.14) | 0.98 (0.76-1.27) | 1.05 (0.81-1.37) | 1.25 (0.88-1.76) |
| **Marital status** |  |  |  |  |  |  |  |  |
| Married/living with spouse | ref. | ref. | ref. | ref. | ref. | ref. | ref. | ref. |
| others | 1.00 (0.72-1.38) | 1.08 (0.78-1.50) | 0.33 (0.09-1.14) | 1.59 (0.17-15.11) | 1.62 (1.13-2.32) | 1.28 (0.92-1.79) | 1.11 (0.79-1.57) | 1.21 (0.78-1.88) |
| **Living arrangement** |  |  |  |  |  |  |  |  |
| Living with family | ref. | ref. | ref. | ref. | ref. | ref. | ref. | ref. |
| Alone | 0.97 (0.66-1.42) | 0.98 (0.79-1.51) | 0.86 (0.25-2.97) | 0.62 (0.05-7.34) | 0.89 (0.58-1.35) | 0.76 (0.51-1.12) | 1.01 (0.67-1.50) | 1.01 (0.61-1.70) |
| **Number of chronic conditions** |  |  |  |  |  |  |  |  |
| 1 | ref. | ref. | ref. | ref. | ref. | ref. | ref. | ref. |
| 2 | 0.99 (0.72-1.37) | 1.09 (0.79-1.51) | 0.80 (0.20-3.18) | 0.14 (0.03-0.74) | 1.35 (0.95-1.92) | 0.78 (0.55-1.10) | 0.85 (0.60-1.21) | 1.16 (0.75-1.78) |
| ≥3 | 0.98 (0.60-1.61) | 1.29 (0.79-2.11) | 0.13 (0.03-0.60) | 0.04 (0.00-0.30) | 2.08 (1.20-3.62) | 0.79 (0.48-1.32) | 0.73 (0.43-1.23) | 0.98 (0.50-1.90) |
| **Number of medications** |  |  |  |  |  |  |  |  |
| 1-2 | ref. | ref. | ref. | ref. | ref. | ref. | ref. | ref. |
| ≥3 | 1.07 (0.75-1.51) | 1.03 (0.72-1.46) | 1.03 (0.27-3.91) | 3.10 (0.59-16.17) | 0.83 (0.57-1.20) | 1.60 (1.11-2.31) | 1.53 (1.05-2.21) | 1.03 (0.64-1.64) |
| **Self-rated health** |  |  |  |  |  |  |  |  |
| Excellent/good | ref. | ref. | ref. | ref. | ref. | ref. | ref. | ref. |
| Fair/poor | 0.77 (0.53-1.13) | 0.69 (0.47-1.01) | 0.14 (0.05-0.37) | 0.25 (0.06-1.04) | 0.40 (0.27-0.59) | 0.68 (0.45-1.02) | 0.46 (0.29-0.72) | 1.61 (1.02-2.56) |

**Supplementary Table 3. STrengthening the Reporting of OBservational studies in Epidemiology (STROBE) checklist for cross-sectional studies**

|  | **Item No** | **Recommendation** | **Reported page (s)** |
| --- | --- | --- | --- |
| **Title and abstract** | 1 | (*a*) Indicate the study’s design with a commonly used term in the title or the abstract | 3 |
|  |  | (*b*) Provide in the abstract an informative and balanced summary of what was done and what was found | 3-4 |
| **Introduction** | | |  |
| Background/rationale | 2 | Explain the scientific background and rationale for the investigation being reported | 5 |
| Objectives | 3 | State specific objectives, including any prespecified hypotheses | 6 |
| **Methods** | | |  |
| Study design | 4 | Present key elements of study design early in the paper | 7 |
| Setting | 5 | Describe the setting, locations, and relevant dates, including periods of recruitment, exposure, follow-up, and data collection | 7 |
| Participants | 6 | (*a*) Give the eligibility criteria, and the sources and methods of selection of participants | 7 |
| Variables | 7 | Clearly define all outcomes, exposures, predictors, potential confounders, and effect modifiers. Give diagnostic criteria, if applicable | 8 |
| Data sources/ measurement | 8* | For each variable of interest, give sources of data and details of methods of assessment (measurement). Describe comparability of assessment methods if there is more than one group | 7-8 |
| Bias | 9 | Describe any efforts to address potential sources of bias | 9 |
| Study size | 10 | Explain how the study size was arrived at | 7 |
| Quantitative variables | 11 | Explain how quantitative variables were handled in the analyses. If applicable, describe which groupings were chosen and why | 9 |
| Statistical methods | 12 | (*a*) Describe all statistical methods, including those used to control for confounding | 9 |
|  |  | (*b*) Describe any methods used to examine subgroups and interactions | NA |
|  |  | (*c*) Explain how missing data were addressed | 9 |
|  |  | (*d*) If applicable, describe analytical methods taking account of sampling strategy | NA |
|  |  | (*e*) Describe any sensitivity analyses | NA |
| **Results** | | |  |
| Participants | 13* | (a) Report numbers of individuals at each stage of study—eg numbers potentially eligible, examined for eligibility, confirmed eligible, included in the study, completing follow-up, and analysed | 10 |
|  |  | (b) Give reasons for non-participation at each stage | NA |
|  |  | (c) Consider use of a flow diagram | NA |
| Descriptive data | 14* | (a) Give characteristics of study participants (eg demographic, clinical, social) and information on exposures and potential confounders | 10 |
|  |  | (b) Indicate number of participants with missing data for each variable of interest | 10 |
| Outcome data | 15* | Report numbers of outcome events or summary measures | 10 |
| Main results | 16 | (*a*) Give unadjusted estimates and, if applicable, confounder-adjusted estimates and their precision (eg, 95% confidence interval). Make clear which confounders were adjusted for and why they were included | 11 |
|  |  | (*b*) Report category boundaries when continuous variables were categorized | NA |
|  |  | (*c*) If relevant, consider translating estimates of relative risk into absolute risk for a meaningful time period | NA |
| Other analyses | 17 | Report other analyses done—eg analyses of subgroups and interactions, and sensitivity analyses | NA |
| **Discussion** | | |  |
| Key results | 18 | Summarise key results with reference to study objectives | 12-13 |
| Limitations | 19 | Discuss limitations of the study, taking into account sources of potential bias or imprecision. Discuss both direction and magnitude of any potential bias | 14 |
| Interpretation | 20 | Give a cautious overall interpretation of results considering objectives, limitations, multiplicity of analyses, results from similar studies, and other relevant evidence | 12-13 |
| Generalisability | 21 | Discuss the generalisability (external validity) of the study results | 15 |
| **Other information** | | |  |
| Funding | 22 | Give the source of funding and the role of the funders for the present study and, if applicable, for the original study on which the present article is based | 22 |
